# The Effects of Yoga on Bipolar Disorder: A Systematic Review

**DOI:** 10.7759/cureus.27688

**Published:** 2022-08-04

**Authors:** Marie Jean, Muaaz Umair, Pratyusha Muddaloor, Michelle Farinango, Akhil Ansary, Amulya Dakka, Zahra Nazir, Humaira Shamim, Gokul Paidi, Safeera Khan

**Affiliations:** 1 Psychiatry, California Institute of Behavioral Neurosciences & Psychology, Fairfield, USA; 2 Research, California Institute of Behavioral Neurosciences & Psychology, Fairfield, USA; 3 Internal Medicine, California Institute of Behavioral Neurosciences & Psychology, Fairfield, USA; 4 Clinical Sciences, St. Martinus University Faculty of Medicine, Willemstad, CUW; 5 Dermatology, California Institute of Behavioral Neurosciences & Psychology, Fairfield, USA; 6 Family Medicine, California Institute of Behavioral Neurosciences & Psychology, Fairfield, USA

**Keywords:** alternative therapry for bipolar, bipolar disorder treatment, major depression disorder, mood disorder, mania, depression, bipolar disorder, yoga

## Abstract

Bipolar disorder (BD) is a mood disorder characterized by severe mood swings and or periods of depression. This study examined the role that practicing yoga has on the symptoms of BD. One of the main goals was to identify if patients with BD believe that yoga is a viable treatment option. Six research databases were searched using the keywords "yoga" AND "therapy" AND "BD" AND "bipolar depression." Articles published in 2005 and later were included in the search. After duplicates were removed, and inclusion and exclusion criteria were applied, five articles were analyzed and included in this literature review. Results of this review indicate that yoga has been shown to be associated with both benefits and risks for the treatment of BD. Studies have shown that yoga might relieve some symptoms of BD and depression. However, due to the lack of research on the impact of yoga on BD and the small number of studies included in this review, results should be approached with caution. Overall, yoga was well-tolerated in the studies reviewed in this article. Yoga may relieve the symptoms of depression. Future research should analyze the long-term impact of yoga on bipolar depression. Yoga instructional standards should also be considered.

## Introduction and background

An estimated 9.7% of adults in the United States will suffer from a mood disorder during the course of a year [1]. Major depression and bipolar disorder (BD) are two of the most common types of mood disorders, which are characterized by emotional impairment [2]. Dysthymia, unipolar depression, unipolar mania, and cyclothymic disorder are variations of these conditions.

Overview

BD is a mental disorder that causes unstable changes in mood and interferes with daily life activities. A person with BD may suffer from depression, mania, or mixed symptoms that cause distress [3]. During manic episodes, a person may have feelings of grandiosity, excessive talking, and racing thoughts, and these uncontrollable elevated moods may lead to reckless, and dangerous actions [4]. Mania, or hypomania (the subsyndromal counterpart of manic symptoms), is a time of excessively high, expansive, or irritated mood that occurs repeatedly in people with BD [5]. Individuals with BD may exhibit wide ranges in symptom severity and frequency, as well as the duration of and degree of recovery between episodes. During depressive periods, a person may have difficulty sleeping, lack interest in things they once loved, and lose motivation to work and care for themselves. A person may become so overloaded with negative thoughts that they become suicidal [4].

Bipolar disorder management

BD is usually diagnosed in the late juvenile or early adulthood years; however, the management of BD is typically a lifelong process [4]. Scientists are trying to figure out what treatment options work best for BD. Because, without proper treatment, BD typically intensifies. But with a good and comprehensive treatment plan, many people with BD lead healthy and productive lives. Currently, BD is managed using multiple resources such as psychotherapy, pharmacology, patient education, and healthy habits such as exercise [6]. These methods of treating BD encompass and target possible causes of the illness, such as genetics, stress, and brain structure and function.

Systematic review research article purpose

This systematic review evaluates yoga as a potential treatment for BD. It examined if yoga therapy is a beneficial treatment approach to managing BD and what role it should play in the management of BD. Results in BD have generally been based on evaluations of objectively measurable clinical information, such as rates of relapse and degrees of symptom reduction on clinician-rated assessment measures. Evaluating outcomes based on activities of daily living or other functional measures has recently become an additional important measure to consider. The capacity of a person to carry out everyday activities and participate in interactions with others in ways that are pleasurable for both the individual and others around them, as well as meeting the requirements of the community in which they live, is referred to as their "psychosocial functioning" [7]. Quantitative research approaches have been employed in the majority of studies on psychosocial functioning in BD [8-10], but not all [11]. Few studies have studied the psychosocial functioning of healthy or inter-episode persons in these quantitative investigations; most have focused on individuals who are symptomatic or suffering a mood episode [12]. BD patients' self-management tactics have only been explored in one prior qualitative research, to our knowledge [13]. A "keep well" idea and "ways to remain well" emerged as the study's two key topics. Treatment access support and stay-well plans were among the most common methods of staying well after diagnosis, as were acceptance of the diagnosis, education on mindfulness, identification of triggers and warning indicators, stress management, sleep hygiene, and other lifestyle modifications. However, despite Russell and Browne's (2005) study initial data value, it was marred by significant methodological shortcomings. With no objective evaluation or clinical information obtained, participants of the research self-identified as being in good health throughout the course of a two-year period. By conducting a qualitative investigation of the self-management methods employed by high-functioning individuals with BD, we hoped to add to this body of work. It was our goal to avoid some of the flaws of Russell and Browne's (2005) study in our qualitative research by conducting rigorous qualitative analysis techniques and using a carefully screened sample of participants, as well as collecting quantitative data on symptoms, psychosocial functioning and quality of life (QoL). One of the main goals of this paper is to describe the categories of self-management strategies that BD patients who are "high functioning" identify as effective, and to examine the clinical implications of these findings in light of existing strategies in adjunctive psychosocial interventions for BD.

## Review

Research Methodology

In accordance with the Preferred Reporting Items for Systematic Reviews and Meta-Analyses (PRISMA) standards, this systematic review was entered [14].

The following combination of search phrases was used for all databases included: “yoga” AND “therapy” AND “bipolar disorder” AND bipolar depression.” Articles that have been published since 2005 were all included. Articles were searched from PubMed, Emerald, ScienceDirect, SAGE Publications, Wiley, and Google Scholar. 

The following inclusion and exclusion criteria used in this review are presented in (Table 1) below.

**Table 1:** Inclusion and Exclusion Criteria

Inclusion Criteria	Exclusion Criteria
Peer reviewed	Non-peer reviewed
Articles mention the application of yoga for patients suffering from depression	Include other interventions
Meet diagnostic criteria	Does not meet the diagnostic criteria
Published in 2005 or after	Published prior to 2005

Studies whose abstracts indicated that they provided empirical research and information about the use of yoga as a treatment for depression were given preference. The articles utilized for the overall analysis were required to describe how the integration is carried out, illustrate the efficacy of the integration, or outline both aspects.

**Figure 1:**
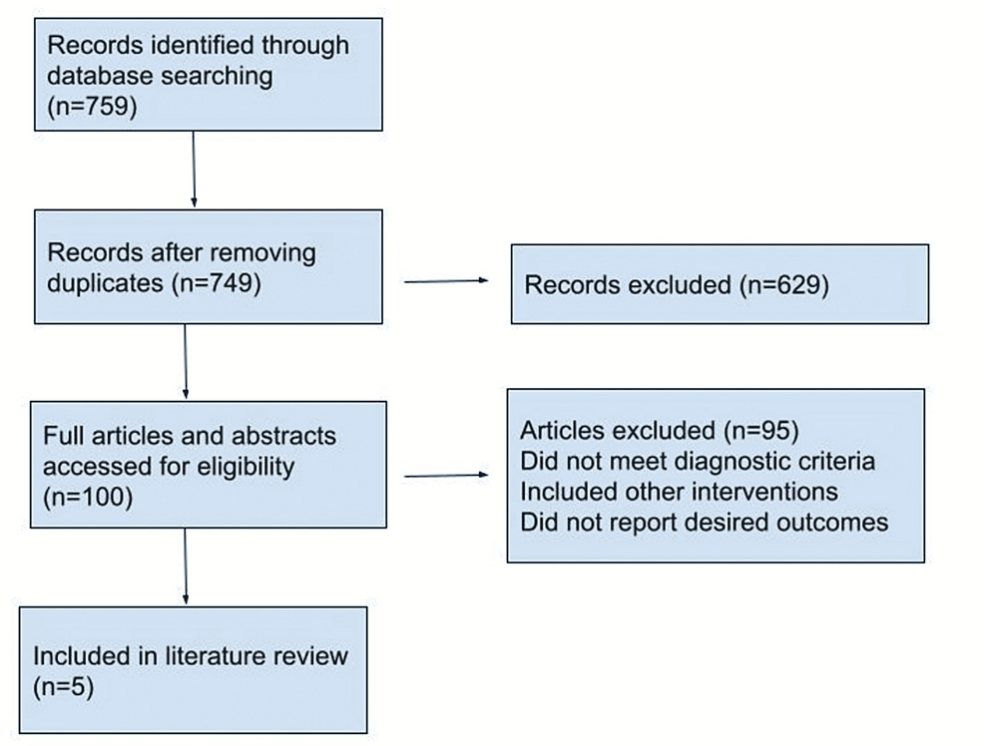
Identification and Selection of Studies for the Review Identification and selection of studies for the review. Adapted from Page et al. [14]

Out of 759 research articles that were checked according to the keywords used, only five were included in this systematic review research paper (Figure 1).

The details from the articles taken for the review are presented in Table 2 below:

**Table 2:** Summary of Articles in the Review BD, bipolar disorder; RCTs, randomized controlled trials; MADRS, Montgomery-Asberg Depression Rating Scale; QoL, quality of life; CAM, complementary and alternative medicine; SAMe, S-adenosyl-L-methionine.

Author	Title	Background	Method	Result	Conclusions
Uebelacker et al. (2014) [15]	Self-Reported Benefits and Risks of Yoga in Individuals with BD	Although hatha yoga is often advocated for patients with BD and there is evidence that it may reduce depression, there is no published research on yoga’s benefits and associated risks. Thus, the goal of this research was to investigate the risks and benefits of yoga in persons with BD.	Yoga practitioners diagnosed with BD (N=109) were invited to participate in an internet survey that included demographic and clinical questionnaires as well as open-ended inquiries regarding yoga practice and its effects.	A total of 86 participants provided enough information for analysis, with 70 of them meeting the criteria for a lifetime history of mania or hypomania. Hatha and vinyasa were the most popular types of yoga. Emotional impacts, such as decreased anxiety, good cognitive effects (e.g., acceptance, attention), or pleasant physical effects (e.g., weight loss, increased energy) were the most reported outcomes for participants. Yoga was seen as a major life-altering experience by several of the participants. Yoga’s most prevalent side effect was pain or injury. Five respondents described situations in which yoga enhances agitation or manic symptoms. Also, five respondents described times when yoga increased melancholy or lethargy.	58 participants believe that yoga is good for their mental health at least some of the time. However, yoga does not come without its own set of risks. Yoga may be able to help those with BD by providing an additional therapy option.
Cramer et al. (2017) [16]	A Systematic Review of Yoga for Major Depressive Disorder	The goal of this review was to examine the effectiveness and safety of yoga therapies in the treatment of individuals with severe depressive illness.	Through the end of 2016, MEDLINE, Scopus, and the Cochrane Library were all checked. Trials comparing the effects of yoga on patients with major depressive disorder with those of inactive or active control groups were eligible for inclusion in the review. Remission rates and depression severity were two of the study’s primary objectives. Secondary results were anxiety and adverse events.	There were 240 participants in seven RCTs analyzed. Most of the RCTs had an unclear risk of bias. There were no changes in depression severity between the aerobic exercise and control groups during the short or medium term. Yoga was shown to have more severe short-term depression symptoms than electro-convulsive therapy. However, there was no difference in remission rates between the two treatments. When yoga was compared to antidepressant medications, there were no short-term changes between the groups. When yoga was compared to attention-control therapies or anti-depressant medication alone, conflicting data were observed in the studies. It was shown that just two RCTs assessed treatment-related side events and those two RCTs did not identify any adverse events.	In this study, there were some indications of yoga yielding favorable benefits beyond placebo and similar results to evidence-based therapies. However, due to methodological issues and an indeterminate risk-benefit ratio, one can’t propose yoga as an additional therapy for severe depressive illness. Non-inferiority designs and larger and more powerful RCTs are required.
Ravindran et al. (2021) [17]	Breathing-Focused Yoga as Augmentation for Unipolar and Bipolar Depression: A Randomized Controlled Trial	Even after receiving treatment for their depression, many patients still suffer from chronic depressive symptoms. As a preferred alternative or supplemental therapy, some individuals turn to complementary therapies, such as yoga. While there is some evidence that yoga may help with depression, this hasn’t been well studied, especially in the case of bipolar depression. Manualized breathing focus yoga was compared to psychoeducation as an adjunct to medicine in the treatment of depression in both unipolar and bipolar patients.	72 outpatients with unipolar or bipolar depression were given two 8-week therapies as an adjunct to their existing first-line antidepressants and mood stabilizers in a single-blind crossover design. The Montgomery-Asberg Depression Rating Scale (MADRS) was used as the major outcome measure. Because of the large number of individuals who left the trial after the crossover at week 8, the emphasis of the analysis was on the comparison between yoga and psychoeducation during the study’s first eight weeks. The treatment sessions were provided two times per week and were 1.5 hours each session.	Participants' attendance was similar in both the yoga and psychoeducation groups. After eight weeks of yoga, the MADRS showed a substantial decrease in depression symptoms. Intervention groups did not show a statistically significant change in MADRS scores. Self-reported measures of depression and well-being showed similar improvements over time.	In addition to pharmacotherapies, yoga and psychoeducation may help alleviate some of the symptoms of both unipolar and bipolar depression. Long study periods and group sessions in the classroom may make it more difficult for participants in this category to succeed. This may be more possible in studies with larger sample sizes, parallel-group designs, and shorter study durations.
Weinstock et al. (2016) [18]	Adjunctive Yoga Versus Bibliotherapy for Bipolar Depression: A Pilot Randomized Controlled Trial	For the treatment of BD, yoga has been advocated, however, there is no documented research. The purpose of this 10-week randomized controlled trial was to test the feasibility, acceptability, and safety of an adjuvant hatha yoga intervention for bipolar depression.	Yoga (n=10) and self-directed bibliotherapy (n=8) were randomized to eighteen persons with bipolar I/II depression both as add-ons to pharmacotherapy for BD. The bibliotherapy group was provided with a personal copy of a self-help book for BD. At least one of the weekly yoga sessions had to be attended for 10 weeks. Change in depression severity was measured by a blind rater after therapy. Additional examinations included questionnaires on symptoms, QoL, and treatment satisfaction.	Nine out of the 10 participants assigned to the yoga group and five out of eight assigned to the bibliotherapy group completed the 10-week endpoint assessment. Within-group analyses of those allocated to yoga demonstrated moderate benefits for improvement in depression symptoms (Cohen’s d = 0.66) and QoL (Cohen’s d = 0.69) despite no significant differences in depression outcomes across groups. As compared to the control group, the yoga group’s manic symptoms intensity stayed low throughout the study; however, manic symptoms increased modestly in the control group (F(1,13) = 7.25, p 0.021). Even though participants only went to 4.80 (SD = 5.12) yoga sessions, their overall positive feeling about the practice was high, and six out of 10 said they continued their yoga practice at home.	Future studies should explore other delivery methods (e.g. the internet) that may not require weekly class attendance.
Andreescu et al. (2008) [19]	Complementary and Alternative Medicine (CAM) in the treatment of BD - a review of the evidence	Patients with mood disorders are increasingly turning to CAM. For the treatment of individuals with bipolar illness, an examination of available scientific information is conducted on the advantages and hazards of CAM.	Because of the scarcity of CAM research involving patients with BD, the majority of the existing information comes from investigations of individuals with major depressive disorder. St. John’s wort (Hypericum perforatum), S-adenosyl-L-methionine (SAMe), and acupuncture have been researched in a series of RCTs in individuals with significant depression. While omega-3 fatty acids have been researched in two controlled studies in BD. In addition, the beneficial effects of yoga on depression have been researched in several RCTs.	St. John's wort seems to be an effective therapy for mild to moderate depression. Depression may also benefit from SAMe. To know the full degree to which these products may lead to manic symptoms, more research is needed. Drug interactions with St. John’s wort are also possible. Omega-3 fatty acids with acupuncture have conflicting results. Research trials report that yoga benefits patients with depression.	CAM therapies including aromatherapy massage and yoga are all but non-existent in the medical literature. If you have bipolar illness, it’s best to wait for further research before using CAM. In addition, patients need to be aware of the potential dangers connected with these therapies.

Discussion

Uebelacker et al. undertook their research to better understand the benefits and hazards of yoga for the treatment of BD. They were effective in attracting their targeted demographic (i.e., individuals with bipolar I or bipolar II disorder who practice a type of Hatha yoga). For some persons with bipolar disease, Hatha yoga may be a strong and effective positive practice; nonetheless, it is not without hazards and should be utilized cautiously, like many other therapies for BD [15].

Yoga seems to be a beneficial practice for certain patients with BD, according to research by Uebelacker et al. The majority of survey respondents reported improved quality of life. However, yoga was not often described as “life-altering” by respondents, although they did express favorable impacts. Meditation and relaxation were the most generally reported beneficial benefits, with enhanced awareness and calmness being the most reported positive outcomes. Several people wrote that yoga helped them do fundamental everyday tasks outside of the yoga studio. This is critical since depression, with its associated lack of desire and withdrawal, is often a primary symptom of BD. In addition to the recognized weight gain associated with some mood stabilizers and antipsychotic medicines, many patients who practiced yoga did so because it is a physical exercise [15]. There was some disagreement among yoga participants regarding whether yoga helped prevent or moderate symptoms, thus it is best used as a supplementary intervention.

However, some studies have identified risks associated with yoga therapy, particularly for those suffering from bipolar illness [15]. Individuals with BD may be particularly affected by extreme activities, such as fast breathing, warm surroundings, or very slow and contemplative techniques. Also, people with bipolar illness who are on commonly prescribed antipsychotic medications may be at greater risk of lithium toxicity and other side effects connected to heat, dehydration, and other heat-related issues if they do hot yoga [15].

Moreover, due to yoga's physical nature, there is a danger of physical harm, as well as a risk of self-criticism through comparison to other students in the class. In research by Uebelacker et al., 11 participants reported physical injury or an escalation of pain. Among yoga participants, the rate of injuries is 1.18 per 1000 yoga hours [20]. The most common area for injuries is the lower extremities, such as the hip, knee, and ankle [20]. That said, the research by Uebelacker et al. is the first stage in a long-term investigation of yoga as an additional treatment for people with BD. Considering the research's limitations, it is important to keep in mind that no structured interview was conducted to confirm the diagnosis of BD in study participants, that the Mood Disorder Questionnaire (MDQ) was used as a screening tool for BD, and that the results of the yoga practice were evaluated exclusively qualitatively [15]. It is important to note that the MDQ has only proven to be moderately sensitive with regard to screening for BD. A further drawback was the selection of a sample of current yoga practitioners as opposed to those who may not have tried yoga. Nevertheless, as a result of this research, we were able to acquire a better knowledge of the possible advantages and potential hazards of Hatha yoga for BD. These findings imply that a pilot study of a yoga intervention for bipolar illness is necessary as the next step in this research path.

Furthermore, depression is a widespread mental illness, affecting 7-21% of people at some point in their lives. It is a primary source of worldwide illness burden, with high morbidity, and occurs in both unipolar and bipolar variants. Combined, these illnesses are predicted to cost the United States more than $200 billion per year in lost productivity and higher health care costs [17]. Pharmacological medicines such as antidepressants or mood stabilizers may be used to treat both unipolar and bipolar depression. However, often patients stop taking their prescriptions because of the negative side effects of these first-line therapies, therefore their limitations are well-known [17]. Psychotherapy, despite its efficacy and popularity, to treat depression, has its own set of obstacles, including lengthy wait lists, high fees, and a shortage of skilled practitioners [17].

About one-third of individuals with depression do not improve despite receiving first-line therapy. Thus, many people are turning to alternative therapies to relieve their depression. Alternative and complementary approaches to the treatment of manic-depressive disorders include physical therapies (e.g., yoga, acupuncture), nutritional supplements (e.g., vitamins and minerals), and herbal cures (i.e. plants and plant extracts). Likewise, as a result of a lack of access to traditional therapies, as well as a belief that CAM is safer, more accessible, and less invasive than medications for treating depression, many patients are turning to these alternatives [17]. CAM therapies are used by 57% to 67% of patients with a mood or anxiety disorder, typically in conjunction with conventional therapy and sometimes without their physician's knowledge [19]. For unipolar depression, initial data suggests that CAMs, including yoga, may be beneficial, but more substantial research is needed. For bipolar depression, there is even less research on the advantages of CAMs.

Yoga as a treatment for depression

India’s centuries-old socio-cultural tradition of yoga, which is now widely acknowledged as a secular practice across the globe, was the inspiration for its creation. Asanas (postures), pranayamas (breathing exercises), and dhyana (meditation) are the three basic components of modern western yoga disciplines. The three components of yoga are generally present in all forms of yoga, but the emphasis on one or the other may vary. 

RCTs and open studies have demonstrated yoga to be useful in the treatment of unipolar depressive disorder (UDD). Studies have shown that doing yoga, either alone or in concert with other treatments, may alleviate the symptoms of mild to moderate depression. Suicidal thoughts may be alleviated by this treatment, according to preliminary research [17]. In general, yoga is well tolerated, with just a few moderate, uncommon, and related to physical fitness-side effects described. Yoga’s involvement in the therapy of bipolar depression, on the other hand, has received less attention. According to preliminary findings, yoga has a positive effect on mental and physical health, and this must be studied further [21].

Although the first results are encouraging, the lack of research and methodological restrictions, such as small sample numbers, variability in clinical measures, blinding procedures, and patient selection, limit the reliability and generalizability of these findings. In addition, many yoga publications do not include the most important aspects of yoga in their instructions, making it difficult for practitioners to know what to expect. This is a serious flaw, considering the wide variety of physiological and psychological effects and outcomes associated with various forms of yoga. RCTs also often lacked comparators or tested yoga against less stringent control settings (e.g., no treatment, waitlist). Comparative investigations of established therapies are few and far between [16].

Consequences of yoga's antidepressant effects and yoga's mechanisms of action

If yoga's therapeutic benefits are related to stabilization of the hypothalamic-pituitary-adrenal axis activity and autonomic nervous system functioning following stress response, then the mechanisms by which this occurs are yet to be known [17]. The ability to regulate one's breath (known as pranamaya) is thought to be crucial in the return of the body to normal after a stressful event. When practicing yoga regularly, researchers have shown that a number of neuroendocrine and autonomic abnormalities linked with depression, such as hypercortisolemia, and heart rate variability may be normalized [17]. Vagal afferents to autonomic, neuroendocrine, and limbic circuits are thought to be activated by yogic breathing in neurophysiological models, which may help regulate emotions and stress [22].

Limitations

As a result of the limited number of studies included, results from the control and diagnostic category subgroup analyses should be treated with care. Only a limited number of papers were available for each kind of control group in this study, thereby restricting the comparison of yoga with other therapies for depressed symptoms in this review. In addition, the primary studies included in this review have several limitations, including non-standard reporting of intervention protocols, lack of intervention description (e.g., the type or type and components of yoga prescribed), inadequate blinding of assessors, inadequate follow-up, and lack of intention to treat analysis.

## Conclusions

What impact does yoga have on relieving BD symptoms? A closer examination of yoga in contrast to traditional or purposely non-mindful exercise may help discover the most beneficial aspects of yoga practice. As a result, we were unable to draw conclusions on the long-term benefits of yoga on BD symptoms. In our study, we observed that getting to weekly yoga classes may be difficult for some yoga participants. In the design of future yoga therapies aimed at reducing depressive symptoms in patients with BD, this is a significant discovery. Rather than requiring weekly yoga class attendance, interventions should focus on designing an at-home yoga program that can be monitored. It would be worthwhile to look into how long the benefits last and if some varieties of yoga are more effective than others. Also, qualification in yoga practice, on the other hand, has yet to be shown to influence results. As of now, yoga teacher training and education courses are neither regulated nor standardized, nor are they approved by any higher education organizations. In clinical populations with chronic and complicated illnesses that need an in-depth knowledge of pathology as well as psychopathology, this may have ramifications. Exercise and yoga professionals that work with this demographic should seek extra mental health first aid training and upskilling. Yoga practitioners who offer interventions have a variety of training and experience that should be included in future studies to see how it affects intervention results and efficacy.

Overall, this study found that yoga is relatively safe and effective for those with BD. There were no significant differences in attendance or dropout rates between yoga and control groups, with some studies indicating stronger adherence to yoga than in the control condition. Accessible yoga treatments in both inpatient and outpatient mental health facilities to overcome the frequent obstacle of difficulty getting to classes are expected to have favorable impacts on symptoms of BD and increase physical activity levels.
